# Switchable DNA wire: deposition-stripping of copper nanoclusters as an “ON-OFF” nanoswitch

**DOI:** 10.1038/srep19515

**Published:** 2016-01-19

**Authors:** Xiaoli Zhu, Siyu Liu, Jiepei Cao, Xiaoxia Mao, Genxi Li

**Affiliations:** 1 Laboratory of Biosensing Technology, School of Life Sciences, Shanghai University, 200444, People’s Republic of China; 2 State Key Laboratory of Pharmaceutical Biotechnology, Department of Biochemistry, Nanjing University, 210093, People’s Republic of China

## Abstract

Today, a consensus that DNA working as a molecular wire shows promise in nanoscale electronics is reached. Considering that the “ON-OFF” switch is the basis of a logic circuit, the switch of DNA-mediated charge transport (DNA CT) should be conquered. Here, on the basis of chemical or electrochemical deposition and stripping of DNA-templated copper nanoclusters (CuNCs), we develop an “ON-OFF” nanoswitch for DNA CT. While CuNCs are deposited, the DNA CT is blocked, which can be also recovered after stripping the CuNCs. A switch cycle can be completed in a few seconds and can be repeated for many times. Moreover, by regulating the amount of reagents, deposition/stripping time, applied potential, etc., the switch is adjustable to make the wire at either an “ON-OFF” state or an intermediate state. We believe that this concept and the successful implementation will promote the practical application of DNA wire one step further.

Beyond the role as genetic information carrier, DNA has also attracted great interests of physicists, materials scientists, and chemists for its specific structure and physico-chemical properties^1^,^2^,^3^,^4^. In 1960s, Eley and Spivey first proposed the concept of DNA conductivity^5^. Since then, a large number of studies have confirmed that electron might indeed transport through DNA chains, a phenomenon referred to as DNA-mediated charge transport (DNA CT)^6^,^7^,^8^. By means of electrochemical techniques, time-resolved fluorescence, scanning tunnelling microscopy (STM), etc., deep understanding of DNA CT has also been achieved in recent decade. For example, it has been clearly interpreted that it is the overlapping π orbitals of stacked bases that function as the path for the conductivity. The rate of DNA CT is proven to be very fast (at a level of picosecond time)^9^. Furthermore, distance dependence and sequence dependence of DNA CT have also been investigated^10^,^11^,^12^. Though a thorough understanding of DNA CT is still lacking, these efforts have promoted DNA as a promising nanowire for the construction of nanoscale logic circuits^13^.

Some other advantages, e.g. high yield synthesis, near-unity purification, and nanoscale self-organization, have also made DNA a good alternative for use as a nanowire. However, to implement the practical application, two main problems have to be confronted. One is that consistent electrical properties along a considerable distance of DNA wire should be guaranteed. As long as the array of DNA bases is well stacked, DNA CT should, in theory, occur efficiently through hundreds of base pairs, and likely much farther. However, electrochemical observation of DNA CT is limited to a range of a few nanometers (a dozen base pairs) for a long time^14^. Until recently, Barton group have just raised the ceiling to 34 nm (100 base pairs)^7^. It is still far from the demands of semiconductor industry, and more efforts need to be exerted in this aspect. Another problem is the rational switch of the DNA CT path, since the “ON-OFF” switch is the basis of a logic circuit. Though single base-pair mismatch may lead to the significant attenuation of the CT^15^, it is almost impossible to manipulate a certain base-pair of a DNA wire at will and thus to switch the DNA between conducting and insulating.

In this work, we, for the first time, develop a simple but effective method to switch the DNA CT. DNA-templated copper nanoclusters (CuNCs) are synthesized as an “ON-OFF” nanoswitch. Chemical and electrochemical control of the nanoswitch is realized, and may be applied for different purpose. For electrochemical switch, DNA can turn from conducting to insulating in 2 s, and return back to conducting within 1 s. The process can be recycled for quite many times; and the whole process can proceed under rational control. Furthermore, it should also be noted that the DNA CT can be also switched to half-conducting, a tuneable intermediate state between “ON” and “OFF”, by controlling the amount of the DNA-templated CuNCs. Benefited from all the above advantages, this nanoswitch system we have fabricated may promote the practical application of DNA as nanowire.

## Results

**Figure 1:**
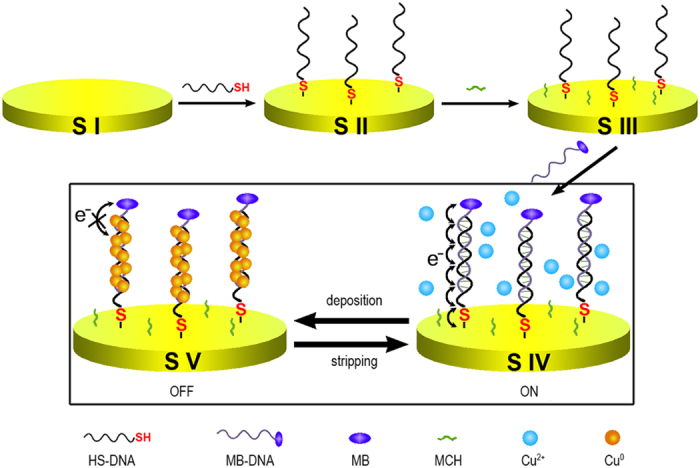
Schematic presentation of the process for the fabrication of the surface tethered DNA wire and the CuNCs-based nanoswitch. The symbol “S” stands for “state”.

Figure 1 shows the principle of the CuNCs-based nanoswitch. A well-established electrochemical sensing system is adopted for the monitoring of the DNA CT^16^,^17^. Briefly, one end of a double-stranded DNA (dsDNA) is connected to a gold electrode, while another end is labelled with a redox active molecule, i.e. methylene blue (MB). CT between the electrode and this signalling molecule may occur through the mediation of dsDNA. To close the CT path, chemical or electrochemical deposition of CuNCs onto the dsDNA is performed. Usually, copper is recognized as a good conductor, and is used for the production of electric wires. However, like some other metallic materials, the rule of size-dependent conductivity also applies to copper^18^. At nanoscale, CuNCs exhibit large electrical resistance. It is thought to be a consequence of the barriers among particle-particle junctions and the small lateral dimensions of the CuNCs (sub-10 nm)^19^. Also because copper is readily available and the reversible redox reaction of Cu^2+^/Cu^0^ is easy to take place, we expect the DNA-templated CuNCs could block and subsequently switch the DNA CT through redox cycles of Cu^2+^/Cu^0^.

**Figure 2:**
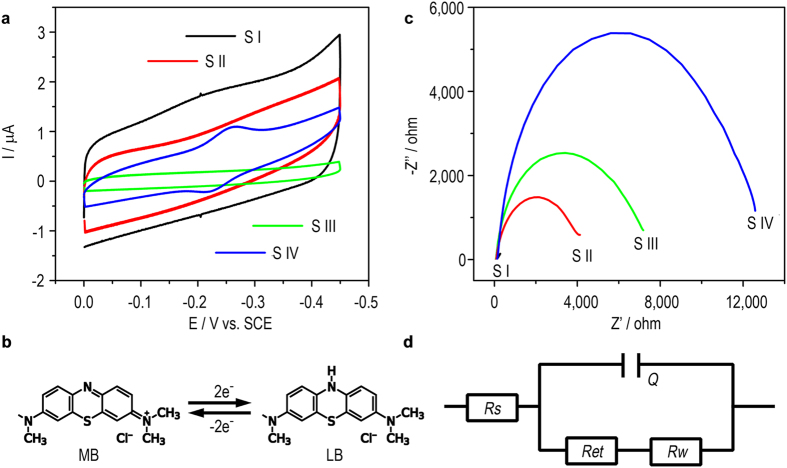
(**a**) Cyclic voltammograms of the electrode under different states, which are corresponding to that shown in Fig. 1; (**b**) The electrochemical redox reaction of MB during the cyclic voltammetric scanning; (**c**) Nyquist diagrams of the electrode under different states; (**d**) Equivalent circuit voltammograms of (**c**), where *R*_*s*_, *Q*, *R*_*et*_ and *R*_*w*_ represent the resistance of the electrolyte solution, the value of constant phase element, the charge-transfer resistance and the Warburg impedance, respectively.

To perform the proof-of-concept design, DNA wire with a surface coverage of 1.5 × 10^13^ molecules/cm^2^ is first fabricated on the working electrode (experimental details and results for the measurements of the DNA coverage are shown in the Supplementary Methods and Figure S1). Electrochemical characterization of the DNA CT as well as the DNA assembly is conducted. As is shown in the cyclic voltammogram of Fig. 2a, only in the presence of the MB-labelled dsDNA (corresponding to state IV, Fig. 1), a quasi- reversible couple of peaks with E^0^ of ca. −0.24 V characteristic of the redox reaction between MB and leucomethylene blue (LB) (Fig. 2b) is observed, suggesting that the DNA CT path has been successfully established. Electrochemical impedance spectroscopy (EIS) is also adopted to characterize the DNA assembly. Figure 2c shows the Nyquist diagrams of different modified electrodes. The diameter of the semicircle represents the interfacial electron-transfer resistance (*R*_*et*_) to [Fe(CN)_6_]^3-/4-^, a commonly used electrochemical active probe. As the electrode is modified from state I to state IV, Larger *R*_*et*_ is observed. The result is reasonable and expected, since that the negative charges and the steric barrier of DNA can hinder the electron transfer between [Fe(CN)_6_]^3–/4–^ and the electrode, resulting in the increase of the *R*_*et*_. In view of the above results, it is concluded that DNA wire is successfully fabricated.

**Figure 3:**
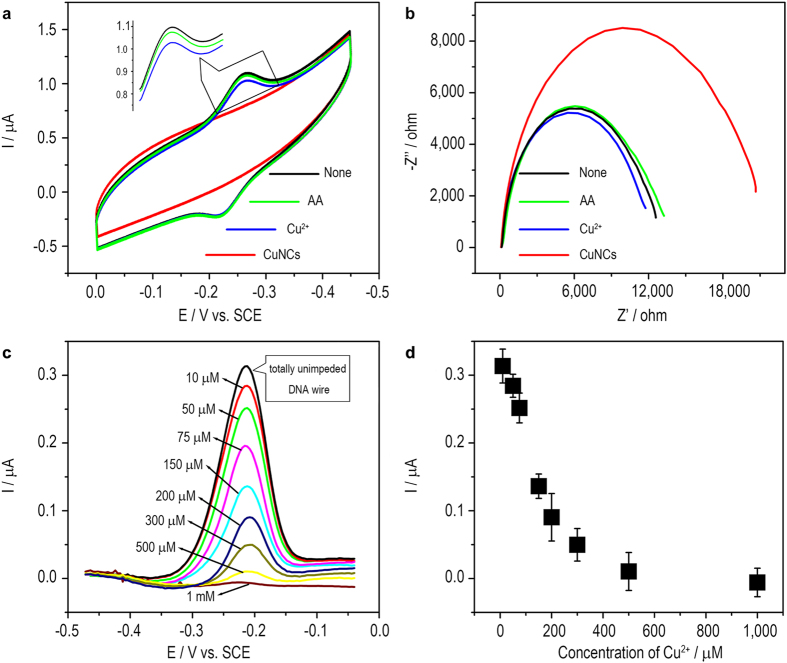
(**a**) Cyclic voltammograms and (**b**) Nyquist diagrams of the DNA wire-tethered electrode before and after the deposition of CuNCs or the treatment with AA or Cu^2+^ ions as control; (**c**) Differential pulse voltammograms of the DNA wire-tethered electrode after the deposition of CuNCs using different concentrations of Cu^2+^ ions; (**d**) The relationship between the differential pulse voltammetry (DPV) peak currents obtained from (**c**) and the concentrations of Cu^2+^ ions.

**Figure 4:**
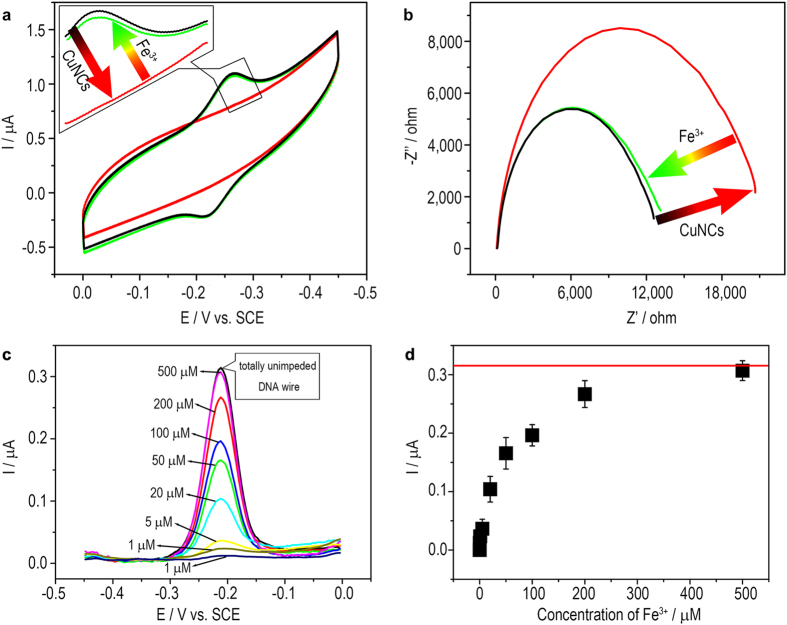
(**a**) Cyclic voltammograms and (**b**) Nyquist diagrams of DNA wire-tethered electrode before and after the deposition of CuNCs and subsequent treatment with Fe^3+^ ions; (**c**) Differential pulse voltammograms of the CuNCs-deposited DNA wire-tethered electrode after the treatment with different concentrations of Fe^3+^ ions; (**d**) The relationship between the DPV peak currents obtained from (**c**) and the concentrations of Fe^3+^ ions. The red horizontal line indicates the current of the unimpeded DNA wire.

**Figure 5:**
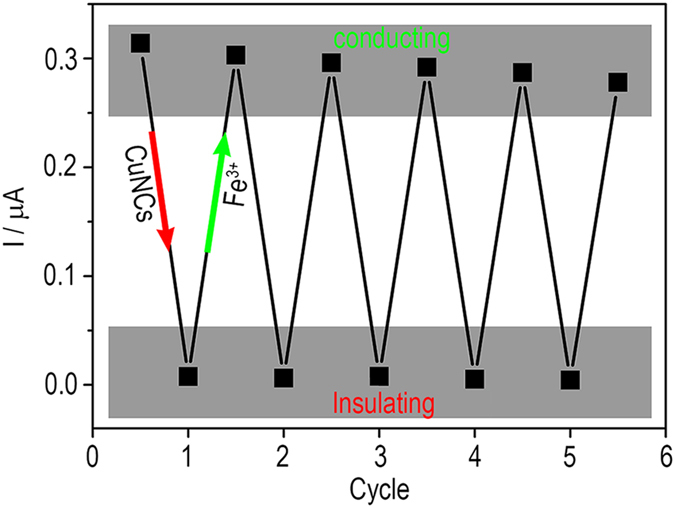
DPV peak currents of the DNA wire-tethered electrode after repeated deposition of CuNCs and subsequent treatment of Fe^3+^ ions in 5 cycles.

Chemical deposition of CuNCs onto the DNA wire is then performed to block the DNA CT. The morphology of the chemically deposited CuNCs is characterized using transmission electron microscope (TEM). Uniform spherical particles with an average diameter of 5.5 nm can be observed (Supplementary Figure S2). While on the surface of an electrode, as is shown in Fig. 3a, the redox peaks of MB disappear if CuNCs are formed, suggesting that the DNA wire is switched OFF. As for the reactants Cu^2+^ and ascorbic acid (AA) respectively working as control, DNA CT retains. EIS results show that the formation of CuNCs further increases the interfacial *R*_*et*_ (Fig. 3b), confirming that the CuNCs have large electrical resistance. The blocking effect is also observed to depend on the amount of the CuNCs. As the increase of the concentration of Cu^2+^ (AA is excess) (Fig. 3c,d), more CuNCs form and consequently hinder the DNA CT till a totally OFF state (state V in Fig. 1). To recover the DNA CT, the CuNCs-deposited DNA-modified electrode surface is treated with Fe^3+^. As is known, the standard electrode potential of Fe^3+^|Fe^2+^ couple is 0.77 V, larger than that of Cu^2+^|Cu^0^ couple (0.34 V). That is, the Cu^0^ of CuNCs can be oxidized to soluble Cu^2+^ by Fe^3+^. As is shown in Fig. 4a, Fe^3+^-treatment does recover the DNA CT. Correspondingly, the *R*_*et*_ is observed to return to a level of unblocked DNA wire (Fig. 4b). The unblocking effect also depends on the concentration of Fe^3+^ (Fig. 4c,d). Treating the blocked DNA wire with 500 μM Fe^3+^ for 30 min is enough to recover the DNA CT to a totally ON state. Thus, a switch cycle of DNA CT is achieved by deposing and subsequently stripping the CuNCs. Several switch cycles are also conducted to show that the DNA CT can be switched between ON (conducting) and OFF (insulating) with a little passivation (Fig. 5).

**Figure 6:**
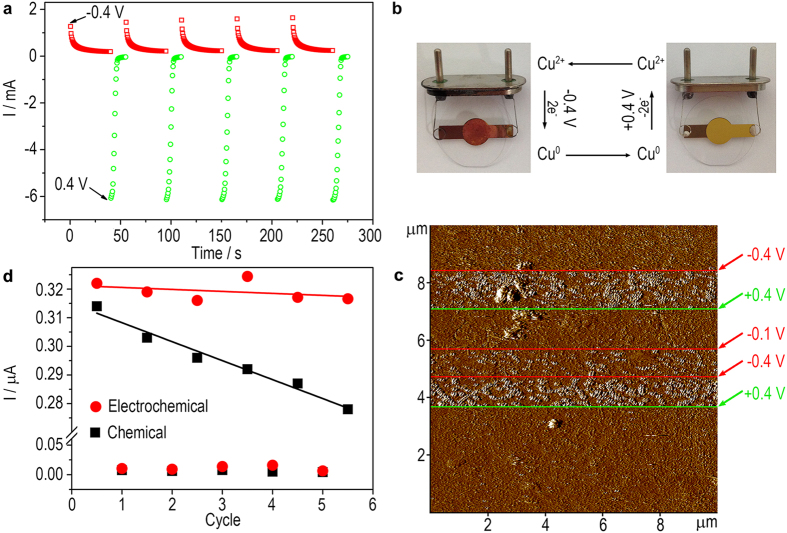
(**a**) I-t curves of the electrochemical deposition (red dots) and stripping (green dots) of CuNCs under −0.4 V and 0.4 V, respectively for 5 cycles; (**b**) The appearance of the electrode after the electrochemical deposition (left) and stripping (right) of CuNCs; (**c**) Real-time AFM imaging of the DNA wire-tethered electrode. The red and green lines indicate the position, where a deposition potential (−0.4 V) and a stripping potential (0.4 V) are applied, respectively; (**d**) DPV peak currents of the DNA wire-tethered electrode after repeated electrochemical (red dots) or chemical (black dots) deposition of CuNCs and subsequent treatment of Fe^3+^ ions in 5 cycles.

Though the chemical switch depicted above can work under control, it seems that the efficiency is not so good. The DNA wire should be treated with Cu^2+^/AA and Fe^3+^ by turns; and dozens minutes are required for each cycle. To make the switch faster and more convenient, potential-driven reactions using electrochemical techniques may be an alternative. A working electrode can work as either the electron donor or the receptor of the redox reaction of Cu^2+^|Cu^0^ to replace the chemical reagents AA and Fe^3+^. Because the electrochemical deposition and stripping of Cu^0^ can be orientated only on the DNA-modified electrode through the interfacial electrochemical reaction, the efficiency should be much higher than a chemical reaction that proceeds everywhere in a solution. Experimental results show that electrochemical deposition of CuNCs is achieved under a negative potential (−0.4 V) in 0.1 M H_2_SO_4_ (pH 1). As is shown in Fig. 6a, I-t curves present the real-time deposition of CuNCs (red dots). At the beginning, the deposition current reaches ca. 1.5 mA, suggesting a deposition speed of 7.8 nmol Cu^0^ per second according to the Faraday’s law of electrolysis (Equation(1)):


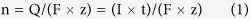


(n: the amount of substance (number of moles), Q: charge, I: current, t: time, F: Faraday constant, z: the valence number of ions of the substance)

The deposition speed slows down rapidly and stabilizes within ca. 30 s, suggesting that saturated CuNCs have been deposited. Under a reverse potential (+0.4 V), the CuNCs are stripped (green dots). The stripping speed starts at as high as 31.6 nmol/s (original stripping current: −6.1 mA) and stabilizes within 7 s. The electrochemical deposition and stripping can be repeated for many cycles without any other operation except the potential switch. Figure 6b shows the appearance of the electrode during the cycles. After deposition, a visible layer of dark red Cu^0^ is observed, while after stripping the electrode returns back to a bright golden appearance. Real-time imaging of the DNA-modified electrode surface using atomic force microscopy (AFM) also confirms the rapid deposition and stripping of CuNCs (average diameter of ~4 nm) through potential switch (Fig. 6c). DPV is then conducted after the electrochemical deposition and stripping to study the DNA CT. As is shown in Fig. 6d, similar to the chemical switch (black dots), electrochemical switch is also achieved (red dots). It is worth noting that besides the drastic shortening of the cycling time (from minutes to seconds), electrochemical switch also has the advantage that the DNA CT in the ON mode can be recovered without apparent passivation after several cycles, which will do favour to repeated switch. Because a strong acid environment is adopted during the electrochemical switch, we have also conducted electrophoresis to study the integrity of the DNA structure. Result shows that the length and the quantity of the DNA sequence keep unchanged after a short treatment with H_2_SO_4_ or the oxidant Fe^3+^ (using in the chemical switch) (experimental details and results are shown in the Supplementary Methods and Figure S3). The result suggests the integrity of the DNA structure during the switch.

**Figure 7:**
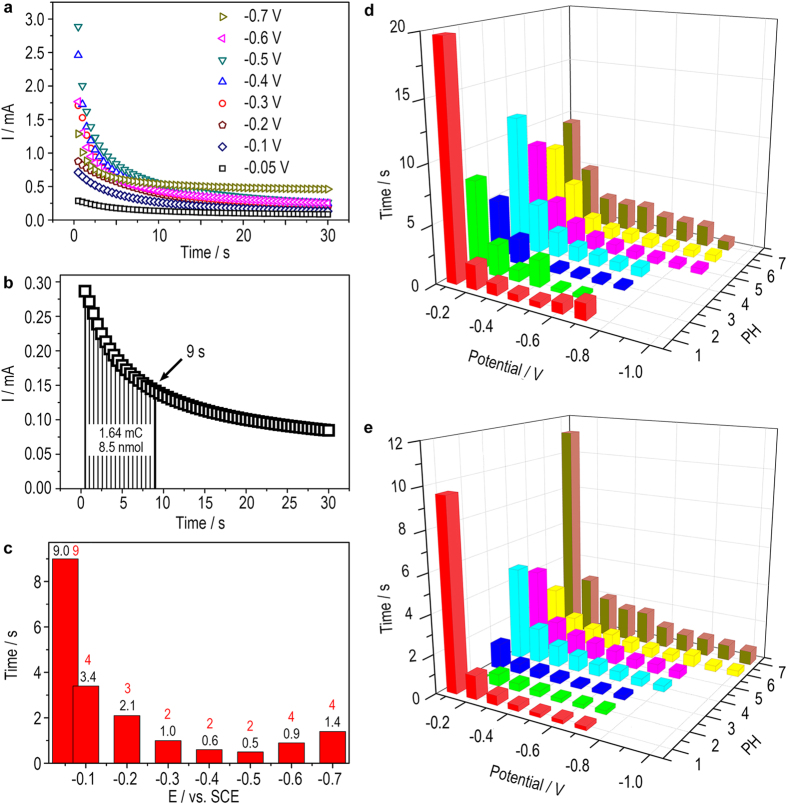
(**a**) I-t curves of the electrochemical deposition of CuNCs under different potentials; (**b**) I-t curves of the electrochemical deposition of CuNCs under −0.05 V. With time, the DNA CT attenuates gradually till a state of totally insulating (9 s); (**c**) Time required for the CuNCs-deposited DNA wire to reach a totally insulating state under different depositing potentials. The numbers marked in black and red are theoretical and measured values, respectively; (**d**,**e**) Theoretical time required for the OFF (electrochemical deposition of CuNCs) and ON (electrochemical stripping of CuNCs) switch of DNA wire, respectively under different potential and pH.

The deposition and stripping of CuNCs are observed to be potential-dependent (Figs 6c and 7a). From Faraday’s law of electrolysis (Equation (1)), the integration of the area under the I-t curves, i.e. the charge, is linear to the actual numbers of moles of the deposited Cu^0^. Using a relatively weak deposition potential (−0.05 V), we are able to slow down the deposition speed thus to study the relation between the DNA CT and the amount of deposited Cu^0^. It is observed that the DNA CT can be totally blocked at ca. 9 s, which corresponds to 1.64 mC and 8.5 nmol of deposited Cu^0^ (Fig. 7b). Therefore, under this circumstance, 9 s is enough to switch the DNA wire OFF; and there is no need to wait for the stabilization of the deposition current just like we have done in Fig. 6. Assuming that this critical amount of Cu^0^ (8.5 nmol) is also applied to other deposition potentials, theoretical critical times for the totally blocking of the DNA CT are obtained, which are basically in agreement with experimental results (Fig. 7c). It is noted that the actual time required (accurate to second) is a little longer than the theoretical time especially for those highly negative potentials. It is probably because some large Cu nanoparticles and aggregates of CuNCs can form under high potential (Fig. 6c), which reduces the blocking efficiency. The above results show that the time required to turn off the DNA CT can be modulated through controlling the applied potential. And like a dimmer switch, the DNA CT can be also switched to an intermediate state by shortening the deposition time. As for the turn-on process, the stripping potential and time can be also modulated to make the DNA CT adjustable. Furthermore, application of electrolytes with different pH also does effect to the deposition and stripping of the CuNCs and consequently the DNA CT, resulting in a multivariate switch. As is shown in Fig. 7d,e, the theoretical time required for the OFF and ON switch respectively under different potential and pH have been calculated from the I-t curves (data not shown).

Finally, we would like to make a discussion. The reason for the blocking of DNA CT by CuNCs has not been addressed in this paper. At present however, one possible mechanism is the electron scattering at the interfaces between the bases of DNA and the CuNCs. It is known that the grain-boundary and surface electron scattering of metal structures increase significantly as their lateral dimensions become comparable to the mean free path of electron (ca. 40 nm in the case of copper), which would lead to a high electron resistivity^20^,^21^. In addition, it has been reported that Cu^2+^ ions could associate with the bases of DNA through the coordination with N or O atoms, e.g. N-7 atom of guanine^22^. Reduction of these coordinated Cu^2+^ ions to Cu^0^ produces CuNCs that very close to the bases. During the CT through the CuNCs-deposited DNA wire, electrons may hop from bases to the interface of CuNCs and then scatter, resulting in the blocking of the DNA CT.

As for the application of DNA CT, though a few reports have addressed some fundamental issues about the practical usage of DNA as wire, relative efforts are still quite lacking. Because the “ON-OFF” switch is the basis of a logic circuit, we think the switch of DNA CT should be solved preceding its application in the coming electronic industry. To our knowledge, this issue is rarely referred ever before. Also in view of the commercial and environmental factors, here we attempt to develop a safe, convenient, and cost-saving method to switch the DNA CT. Inspired by the recent reports of DNA-templated synthesis of CuNCs^23^, and considering that either Cu^0^ or Cu^2+^ are quite familiar in our life, we adopt CuNCs to work as a nanoswitch of DNA wire. The results shown hereinbefore have clearly shown that the CuNCs-based switch system can work well. Other than the advantages, those existing problems of this switch system deserve more attention. First, though rapid response of the switch can be achieved in a few seconds, the response speed is less than satisfactory. Depositing CuNCs onto a targeted certain site but not the entirety of the DNA wire may be a possible solution to elevate the response speed further. Second, as for either the chemical or the electrochemical switch, electron transfer occurs in the redox of Cu^2+^ | Cu^0^, which might affect the CT along DNA wire if integrated together. Using pulse voltage and avoiding the confliction of the potential for copper and signalling molecule might be an effective approach to solve this problem. Finally, it is worth noting that our switch system as well as the DNA CT should be conducted on a solid-liquid interface, which is not similar to a conventional mechanical switch for electric wires. Considering that most reported DNA CT was also carried out on solid-liquid interfaces, we speculate that an adaptable system for DNA CT and switch in a specific form can be developed for practical application in the future.

## Discussion

In summary, we have developed a CuNCs-based nanoswitch for DNA CT. DNA-templated deposition and stripping of CuNCs is achieved through chemical or electrochemical redox reactions. Briefly, Cu^2+^ is reduced to Cu^0^ and deposited onto DNA wire by adding AA (chemical) or a deposition potential (electrochemical). Conversely, the Cu^0^ is oxidized to Cu^2+^ and thereby stripped from the DNA wire by adding Fe^3+^ (chemical) or a stripping potential (electrochemical). Unlike usual conducting copper wires, the DNA-templated CuNCs are found to be insulating and block the DNA CT also. Therefore, repeated deposition and stripping of CuNCs could result in the switch of DNA CT. Dozens minutes are required to complete a switch cycle for the chemical switch, whereas the time required can be shortened to only a few seconds in the electrochemical switch. Both kinds of switch can be cycled over and over, and adjustable to make the DNA wire at either an “ON-OFF” state or an intermediate state. It is the first time that a switch for DNA CT is developed, which also has some advantages such as safe, convenient, and cost-saving. Though there are still some problems to be solved, this concept of switchable DNA wire and the successful implementation will promote the practical application of DNA wire one step further.

## Methods

### Reagents and apparatus

Oligonucleotides were synthesized and purified by Takara Bio Company (Dalian, China). The sequences are as follows:

SH-DNA: 5′-SH-(CH_2_)_6_-TGG CCG TGA CTG GAG ACT GTT A-3′

MB-DNA: 5′-MB-TAA CAG TCT CCA GTC ACG GCC A-3′ (MB: methylene blue)

Tris(2-carboxyethyl)phosphine (TCEP), 6-mercapto-1-hexanol (MCH), ascorbic acid (AA), CuSO_4_·5H_2_O, FeCl_3_·6H_2_O, and some other general reagents were purchased from Sigma-Aldrich. All the reagents were of analytical reagent grade. All solutions were prepared with doubly distilled water (ddH_2_O), which was purified with a Milli-Q purification system (Branstead, USA) to a specific resistance of >18 MΩ cm.

Electrochemical measurements were carried out on a model 660D Electrochemical Analyzer (CHI Instruments, China) with a conventional three-electrode system. Surface morphology was characterized by using an ex situ Agilent 5500 atomic force microscopy (AFM) system. Real-time morphological characterization of the surface of a gold electrode was achieved by connecting the Electrochemical Analyzer with the AFM. Some alterations to the electrolytic cell and the three-electrode system were implemented to obtain electrochemical and morphological signals simultaneously.

### Preparation of surface-tethered DNA wire

A substrate gold electrode was first cleaned with a piranha solution (H_2_SO_4_ : 30% H_2_O_2_ = 3 : 1) for 15 min and then rinsed with ddH_2_O. After that, the electrode was polished to mirror smoothness on a microcloth (Buehler) with gamma micropolish deagglomerated alumina suspension (particle size of ~0.05 μm). Residual alumina powder was removed by sonicating the electrode in ethanol and ddH_2_O for 5 min, respectively. Then, the electrode was electrochemically activated in 1 M H_2_SO_4_ until a stable cyclic voltammogram was obtained. After being rinsed with ddH_2_O and dried with nitrogen, the electrode was ready for functional modification.

To link the electrode with double-stranded DNA wire, the electrode was first allowed to incubate with the SH-DNA (1 μM SH-DNA in a 100 μL TE buffer containing 10 mM Tris, 1 mM EDTA, 0.1 M NaCl, and 1 μM TCEP, pH 7.4) at room temperature for 16 h. The SH-DNA could be tethered onto the electrode surface through an “Au-S” bond. The electrode was then treated with an aqueous solution of 1 mM MCH for 1 h to block those inactive sites and to achieve a well aligned oligonucleotides monolayer. After being rinsed with ddH_2_O, the electrode was further incubated with the MB-DNA (1 μM MB-DNA in a 100 μL PBS buffer containing 10 mM phosphate and 1 M NaCl, pH 7.4) at room temperature for 1 h. Owing to the complementary hybridization between the surface-tethered SH-DNA and the MB-DNA, double-stranded DNA wire was thereby successfully linked onto the electrode surface, and was ready for electrochemical measurements.

### Deposition and stripping of CuNCs onto surface-tethered DNA wire

Chemical deposition of CuNCs was achieved by incubating the modified electrode with an aqueous solution containing 10 μM ~1 mM CuSO_4_ and 2 mM AA for 30 min. The Cu^2+^ could be reduced to Cu^0^ by AA and deposited onto DNA wire preferentially.

As for the stripping, the Cu^0^ was oxidized back to Cu^2+^ by incubating the CuNCs deposited electrode with 0.1 μM ~500 μM FeCl_3_ for 30 min. The residual metallic ions were removed by rinsing the electrode with ddH_2_O.

In electrochemical deposition and stripping, a solution containing 10 mM CuSO_4_ using H_2_SO_4_ to adjust pH from 1 to 7 was adopted as the electrolyte. The modified electrode (working electrode) together with a saturated calomel electrode (reference electrode) and a platinum wire (counter electrode) was employed to constitute a three-electrode system. A constant negative potential (−0.05 V ~ −1.0 V) was applied on the working electrode to reduce Cu^2+^ to Cu^0^. The duration varied in a dozen seconds, which was dependent on the applied potential. As for the stripping, a positive potential (0.05 V ~ 1.0 V) was applied to oxidize Cu^0^ back to Cu^2+^. I-t curves during the electrochemical deposition and stripping were recorded with an interval of 0.5 s.

### Electrochemical measurements

The same three-electrode system as described above was also adopted here. 5 mL PBS (pH 7.0) containing 5.0 mM phosphate, 50 mM NaCl, 4 mM MgCl_2_, 4 mM spermidine, 50 μM EDTA, and 10% glycerol was added into a cell to working as the electrolyte. Before measurements, the electrolyte was thoroughly purged with high purity nitrogen to avoid the interference of dissolved oxygen. A stream of nitrogen was also blown gently across the surface of the electrolyte throughout all the electrochemical measurements. Cyclic voltammetry (CV), differential pulse voltammetry (DPV), and electrochemical impedance spectroscopy (EIS) were conducted. Experimental parameters for CV were as follows: scan rate: 100 mV/s; initial potential: 0 V; final potential: −0.45 V. The same potential range was also adopted in DPV measurements. EIS was recorded in an electrolyte containing 5 mM [Fe(CN)6]^3−/4−^, a commonly used electrochemical active probe. The experimental parameters were as follows: bias potential, 0.224 V; amplitude, 5 mV; frequency range, 0.1 to 10 kHz. The equivalent circuit was fitted using a Nova 1.8 software (Metrohm Autolab B.V.).

## Additional Information

**How to cite this article**: Zhu, X. *et al.* Switchable DNA wire: deposition-stripping of copper nanoclusters as an “ON-OFF” nanoswitch. *Sci. Rep.* **6**, 19515; doi: 10.1038/srep19515 (2016).

## Supplementary Material

Supplementary Information
