# Supplementary material for: Switchable DNA wire: deposition-stripping of copper nanoclusters as an “ON-OFF” nanoswitch
Source: Sci Rep. 2016 Jan 19;6:19515. doi: 10.1038/srep19515 (PMC4725970; doi:10.1038/srep19515)
Supplement: Supplementary Information [file srep19515-s1.pdf]

Switchable DNA wire: deposition-stripping of copper nanoclusters as an "ON-OFF" nanoswitch

Xiaoli Zhu[1], Siyu Liu[1], Jiepei Cao[1], Xiaoxia Mao[1], Genxi Li[1,2*]

[1]Laboratory of Biosensing Technology, School of Life Sciences, Shanghai University, Shanghai 200444, P R China.

[2]Department of Biochemistry and State Key Laboratory of Pharmaceutical Biotechnology, Nanjing University, Nanjing 210093, P R China.

[*]Correspondence should be addressed to Genxi Li. (genxili@nju.edu.cn).

Supplementary Information

Supplementary Methods

Measurement of the DNA coverage. To measure the DNA coverage on the electrode, a routine chronocoulometric method using a redox active cation, i.e. hexaammineruthenium(III) (RuHex), was adopted.[1] The apparatus and the three-electrode setup was used as had been described in the main text. Chronocoulometry experiments were performed in a buffer solution (10 mM tris-HCl, pH 7.4) with or without 50 mM $[Ru(NH_3)_6]^{3+}$. A potential range of 0.2~-0.5 V and a pulse width of 250 ms were adopted. The solution had been saturated with nitrogen prior to measurement to exclude the interference arising from the reduction of dissolved oxygen.

The coverage of DNA on the modified electrode was calculated based on the assumption that the redox active RuHex cation associated with anionic phosphate backbone of DNA.[2-5] The charge corresponding to RuHex electrostatically bound to surface-confined DNA (Q_{ss}) can be calculated from the following equation: $Q_{ss} = Q_{total} - Q_{dl}$ (Q_{total} is the total charge flowing through the electrode and Q_{dl} is the nonfaradaic (capacitive) charges). The relationship between the surface density of electroactive probe (Γ) and Q_{ss} obeys the following equation:

$$\Gamma_{ss} = \frac{z \cdot Q_{SS} \cdot N_A}{m \cdot n \cdot F \cdot A}$$

where n is the number of electrons transferred in the reaction (n = 1), F represents the Faraday constant (coulombs per equivalent), A is the effective surface area of gold

electrode (square centimeters), m is the number of nucleotides in the DNA, z is the charge of the redox molecules, and N_A is Avogadro's number.

According to the above equation and the chronocoulometric results (Figure S1), the DNA coverage is calculated to be 1.5×10^{13} molecules/cm^2.

Polyacrylamide gel electrophoresis. 12% non-denaturing polyacrylamide gel electrophoresis was carried out in a tris-boric acid-disodium EDTA (TBE) buffer at 120 V constant voltage for about 1.5 h. After electrophoresis, the polyacrylamide gel was stained by SYBR Green I, and then imaged using a Gel Doc XR Imaging System.

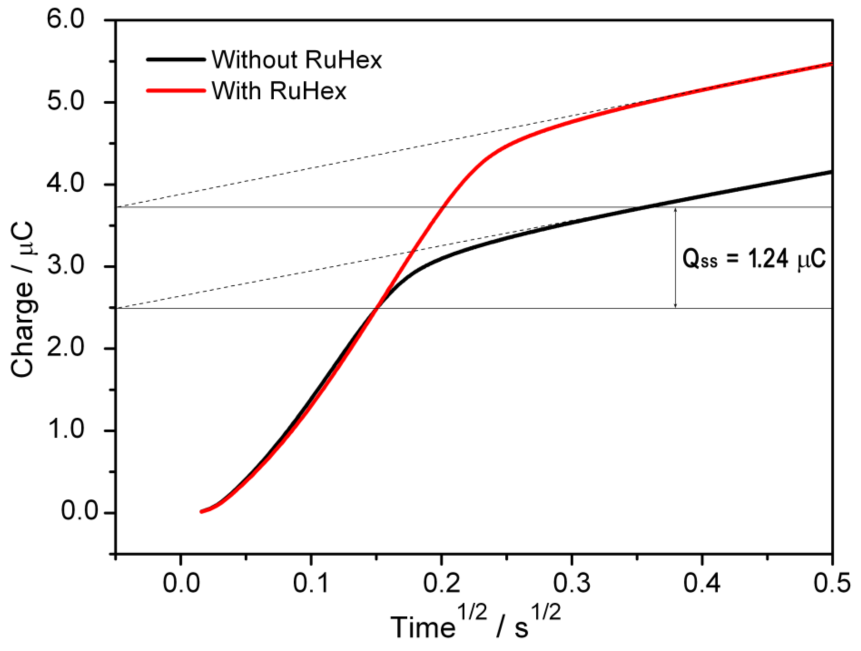

Supplementary Figure S1. Chronocoulometric curves for the DNA modified electrode in the absence (black curve), or in the presence of RuHex (red curve). Q_{total} and Q_{dl} were obtained from the cross points of the tangent line with the Y-axis.

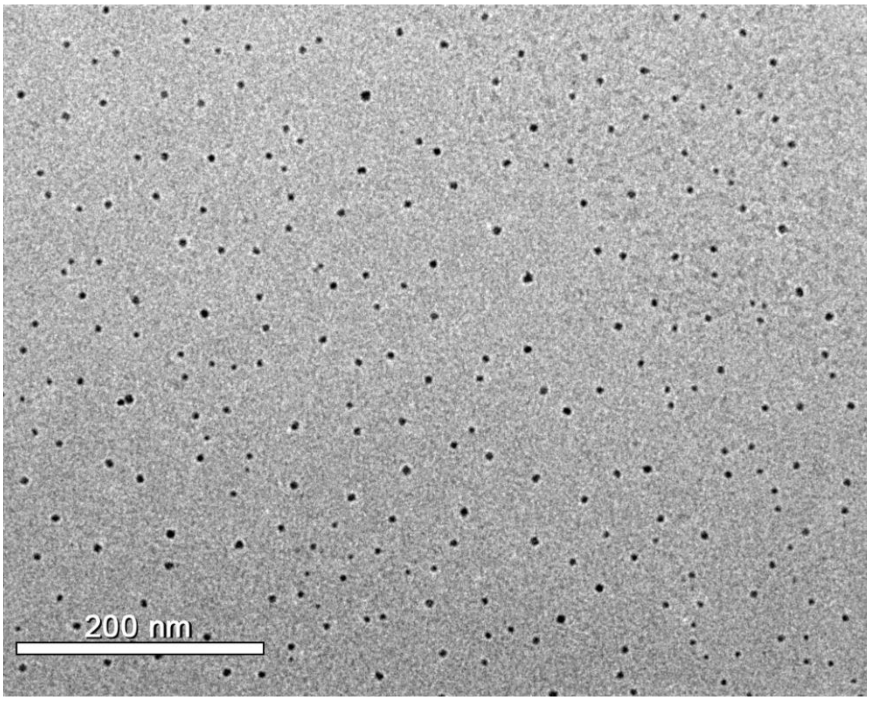

Supplementary Figure S2. TEM image of DNA-templated CuNCs.

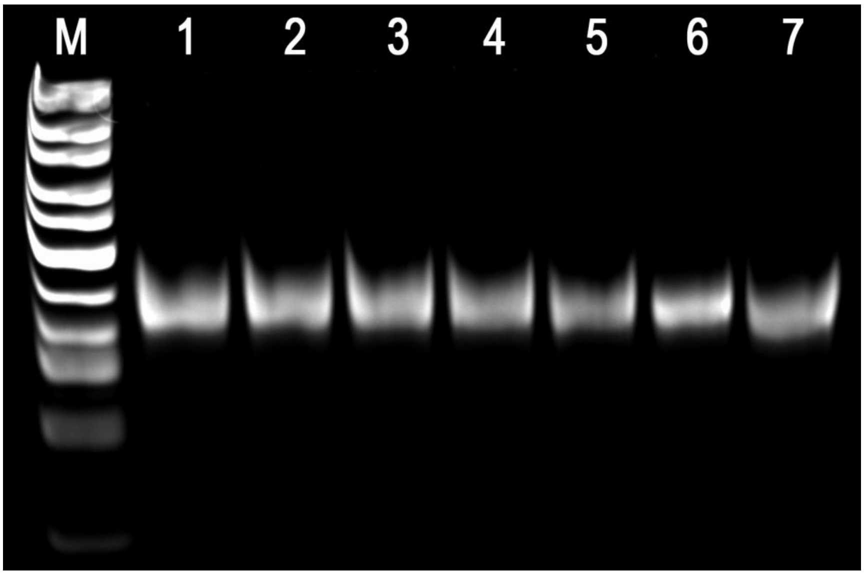

Supplementary Figure S3. Polyacrylamide gel electrophoretic patterns of the double-stranded DNA sequence using in the main text. From left to right: M: 10 bp marker, 1: dsDNA only (1 μM), 2: in the presence of 2 mM ascorbic acid, 3: in the presence of 400 μM Cu^{2+}, 4: in the presence of both ascorbic acid and Cu^{2+}, 5: in the presence of ascorbic acid, Cu^{2+}, and 400 μM Fe^{3+}, 6: in the presence of ascorbic acid, Cu^{2+}, and 0.1 M H_2SO_4, 7: in the presence of ascorbic acid, Cu^{2+}, and 10 mM H_2SO_4. While in the presence of Fe^{3+} or H_2SO_4, the incubation time of dsDNA with Fe^{3+} or H_2SO_4 was 30 min.

References

1. Cao, Y., Zhu, S., Yu, J., Zhu, X., Yin, Y. & Li, G. Protein detection based on small molecule-linked DNA. *Anal. Chem.* **84**, 4314-4320 (2012).

2. Yin, B., Wu, D. & Ye, B. Sensitive DNA-based electrochemical strategy for trace bleomycin detection. *Anal. Chem.* **82**, 8272-8277 (2010).

3. Wu, D., Yin, B. & Ye, B. A label-free electrochemical DNA sensor based on exonuclease III-aided target recycling strategy for sequence-specific detection of femtomolar DNA. *Biosens. Bioelectron.* **28**, 232-238 (2011).

4. Steel, A. B., Herne, T. M. & Tarlov, M. J. Electrochemical quantitation of DNA immobilized on gold. *Anal. Chem.* **70**, 4670-4677 (1998).

5. Lao, R., Song, S., Wu, H., Wang, L., Zhang, Z. & Fan, C. Electrochemical interrogation of DNA monolayers on gold surfaces. *Anal. Chem.* **77**, 6475-6480 (2005).
